# FAM20C‐Mediated Phosphorylation of MEPE and Its Acidic Serine‐ and Aspartate‐Rich Motif

**DOI:** 10.1002/jbm4.10378

**Published:** 2020-06-26

**Authors:** Brian Christensen, Gitte N Schytte, Carsten Scavenius, Jan J Enghild, Marc D McKee, Esben S Sørensen

**Affiliations:** ^1^ Department of Molecular Biology and Genetics Aarhus University Aarhus Denmark; ^2^ Interdisciplinary Nanoscience Center Aarhus University Aarhus Denmark; ^3^ Faculty of Dentistry and Department of Anatomy and Cell Biology McGill University Montreal Quebec Canada

**Keywords:** ASARM, FAM20C, MEPE, PHOSPHORYLATION

## Abstract

Matrix extracellular phosphoglycoprotein (MEPE) is expressed in bone and teeth where it has multiple functions. The C‐terminus of MEPE contains a mineral‐binding, acidic serine‐ and aspartate‐rich motif (ASARM) that is also present in other noncollagenous proteins of mineralized tissues. MEPE‐derived ASARM peptides function in phosphate homeostasis and direct inhibition of bone mineralization in a phosphorylation‐dependent manner. MEPE is phosphorylated by family with sequence similarity 20, member C (FAM20C), which is the main kinase phosphorylating secreted phosphoprotein. Although the functional importance of protein phosphorylation status in mineralization processes has now been well‐established for secreted bone and tooth proteins (particularly for osteopontin), the phosphorylation pattern of MEPE has not been previously determined. Here we provide evidence for a very high phosphorylation level of this protein, reporting on the localization of 31 phosphoresidues in human MEPE after coexpression with FAM20C in HEK293T cells. This includes the finding that all serine residues located in the canonical target sequence of FAM20C (Ser‐x‐Glu) were phosphorylated, thus establishing the major target sites for this kinase. We also show that MEPE has numerous other phosphorylation sites, these not being positioned in the canonical phosphorylation sequence. Of note, and underscoring a possible important function in mineralization biology, all nine serine residues in the ASARM were phosphorylated, even though only two of these were positioned in the Ser‐x‐Glu sequence. The presence of many phosphorylated amino acids in MEPE, and particularly their high density in the ASARM motif, provides an important basis for the understanding of structural and functional interdependencies in mineralization and phosphate homeostasis. © 2020 The Authors. *JBMR Plus* published by Wiley Periodicals, Inc. on behalf of American Society for Bone and Mineral Research.

## Introduction

Matrix extracellular phosphoglycoprotein (MEPE) is highly expressed by osteoblasts, osteocytes, and odontoblasts, and its secretion by these cells plays multiple roles in mineralized tissue biology, including in the regulation of calcification.^(^ ^1^, ^2^ ^)^ Human MEPE consists of 508 amino acids and is a member of the SIBLING protein family (small integrin‐binding ligand, N‐linked glycoproteins) that are also phosphorylated. The five SIBLING proteins are MEPE, dentin matrix protein‐1, bone sialoprotein (BSP), dentin sialophosphoprotein, and osteopontin (OPN).^(^ ^3^, ^4^ ^)^ The SIBLING proteins are intrinsically disordered, yet they share common structural features such as having an integrin‐binding RGD sequence, many potential phosphorylation sites, an abundance of acidic amino acids (Asp and Glu), and a conserved acidic serine and aspartate‐rich motif (ASARM).^(^ ^3^, ^5^ ^)^ The SIBLING proteins play key roles in biomineralization, and their functionalities are highly influenced by posttranslational modifications such as phosphorylation and proteolytic cleavage.^(^ ^3^, ^6^, ^7^, ^8^ ^)^

Several activities have been demonstrated for MEPE in a variety of in vivo and in vitro systems. MEPE‐deficient mice exhibit increased bone formation compared with WT mice.^(^ ^9^, ^10^ ^)^ In other studies, regulation of mineralization by MEPE has been linked to the ASARM motif positioned near the C‐terminus of the protein^(^ ^6^, ^10^, ^11^ ^)^; the ASARM peptide can be released from MEPE by cathepsin B‐catalyzed proteolysis.^(^ ^12^ ^)^ In in vitro studies, the MEPE‐ASARM peptide inhibits de novo hydroxyapatite formation in a cell‐free system,^(^ ^7^ ^)^ and it inhibits mineralization of MC3T3‐E1 osteoblast cultures and bone marrow stromal cell cultures.^(^ ^6^, ^11^ ^)^ Furthermore, in tooth cell culture studies, MEPE‐ASARM impairs odontogenic differentiation and matrix mineralization of dental pulp stem cells.^(^ ^13^ ^)^ MEPE, and particularly the ASARM peptide, have also been implicated in X‐linked hypophosphatemic rickets (XLH).^(^ ^6^, ^14^, ^15^ ^)^ XLH is caused by inactivating mutations of the cell‐surface metalloprotease PHEX (phosphate‐regulating gene with homologies to endopeptidases on the X‐chromosome).^(^ ^16^ ^)^ ASARM peptides derived from the SIBLING proteins, and OPN in particular, are the only known substrates for PHEX.^(^ ^8^, ^11^, ^17^ ^)^ Accumulation of ASARM peptides has been observed in XLH patients and in hypophosphatemic mice leading to mineralization defects in bone and teeth.^(^ ^15^, ^18^ ^)^ In addition, some evidence exists that ASARM peptides upregulate the expression of fibroblast growth factor 23, a circulating factor that inhibits reabsorption of phosphate in the kidneys and intestinal phosphate uptake leading to hypophosphatemia and decreased bone mineralization.^(^ ^2^, ^19^, ^20^ ^)^

Posttranslational modifications of MEPE—such as phosphorylation and proteolytic processing—have significant effects on the biological functions of the protein. The full‐length phosphorylated MEPE protein can act as a promoter of hydroxyapatite formation and growth in a cell‐free in vitro gel‐diffusion system.^(^ ^7^ ^)^ The phosphorylated MEPE‐ASARM peptide is an effective inhibitor of osteoblast cell culture extracellular matrix mineralization, directly binding to and inhibiting hydroxyapatite mineral crystal growth, and having greatly reduced or absent inhibition activity after dephosphorylation.^(^ ^11^ ^)^ Similar to this, in a cell culture model designed to study the underlying mechanisms of dental defects observed in XLH, only phosphorylated MEPE‐derived ASARM peptides inhibited odontogenic cell differentiation and matrix mineralization.^(^ ^13^ ^)^ In terms of the enzymatic degradation of ASARM peptides, PHEX has been shown to rescue the inhibition of mineralization by both phosphorylated MEPE‐ASARM and OPN‐ASARM peptides, and the cleavage sites in ASARM have been determined.^(^ ^11^, ^17^ ^)^ PHEX has also been shown to bind directly to the C‐terminal ASARM motif of MEPE, which protects MEPE and its ASARM from proteolytic degradation.^(^ ^12^, ^21^ ^)^ In this same work, the interaction between MEPE and PHEX was shown to be inhibited by phosphorylated MEPE‐ASARM peptide, but much less effectively by nonphosphorylated MEPE‐ASARM peptide.^(^ ^21^ ^)^ Similarly, the phosphorylated, but not the nonphosphorylated MEPE‐ASARM inhibited the enzyme activity of PHEX.^(^ ^19^ ^)^ Collectively, these studies demonstrate the importance of phosphorylation in the different functions of MEPE, as well as for the MEPE‐ASARM peptide.

Family with sequence similarity 20C (FAM20C) is the main kinase phosphorylating secreted phosphoproteins; it is highly expressed in mineralized tissues.^(^ ^22^, ^23^ ^)^ FAM20C is a serine/threonine kinase with the canonical recognition motif being Ser‐x‐Glu/pSer (phosphoserine), but phosphorylation of secreted proteins has also been reported at a number of other motifs.^(^ ^24^ ^)^ The SIBLING proteins, including MEPE, are well‐established substrates for FAM20C.^(^ ^22^, ^23^ ^)^ MEPE contains many serine residues having FAM20C recognition motifs, and the C‐terminal ASARM motif contains a polyserine stretch that potentially could be targeted by the kinase.^(^ ^25^ ^)^ Heretofore, no studies have investigated the phosphorylation of MEPE and its ASARM peptide, either in vivo or in vitro. Previous work has used synthetic phosphorylated ASARM peptides to provide important functional information,^(^ ^7^, ^11^, ^13^, ^17^, ^19^, ^21^ ^)^; but no information is yet available on actual phosphorylation sites in MEPE. Given this context of the importance of SIBLING protein phosphorylation, we have investigated the phosphorylation pattern for MEPE in an attempt to understand the mechanisms of its (and its peptides) mineralization‐regulating function and interactions with PHEX. Here, we characterize the phosphorylation of MEPE coexpressed with FAM20C in human embryonic kidney 293 cells (HEK293T), and identify a remarkably high total of 31 residues that can be phosphorylated by FAM20C, of which nine are clustered in the ASARM motif.

## Materials and Methods

### Cloning

Human FAM20C cDNA encoding residues 1–584 was amplified by PCR using the forward primer 5′ ACCCAAGCTGGCTAGATATGAAGATGATGCTGGTGCGCCG 3′ and the reverse primer 5′ GTTCGGGCCCAAGCTTCCTCGCCGAGGCGGCTCTG 3′ (LGC Biosearch Technologies, Risskov, Denmark). Amplified cDNA was cloned into the pcDNA3.1/myc His(−)A (Invitrogen, Carlsbad, CA, USA) via NheI and HindIII restriction sites using the In‐Fusion HD cloning technology (TaKaRa Bio, Otsu, France). MEPE‐pcDNA3.1/myc His(−)A (residue 1–525 including signal peptide) was purchased from GenScript (Piscataway, NJ, USA). These constructs encoded human FAM20C and MEPE, both followed immediately by residues KLGP, the *myc* epitope (EQKLISEEDL), residues NSAVD and H_6_. The MEPE‐ (K509A/K515A‐) plasmid was prepared by GenScript by mutation of K^509^LGP to ALGP and EQK^515^LISEEDL to EQALISEEDL in the MEPE plasmid.

The plasmids were propagated in *Escherichia coli* STELLAR competent cells (TaKaRa Bio), and all constructs were verified by sequence analysis. Plasmid DNA for transfection was prepared using PureLink HiPure Plasmid Filter Maxiprep (Invitrogen).

### Protein expression and purification

HEK293T cells were maintained in Dulbecco's modified Eagle's medium with GlutaMAX (Invitrogen), supplemented with 10% FBS and 1% antibiotics (penicillin/streptomycin). HEK293T cells (8 × 10^5^) were seeded in T25‐cell‐culture flasks and grown to confluency of 30% to 70%; at which point, the medium was changed to Opti‐MEM‐reduced serum medium supplemented with 5% FBS and 1% penicillin/streptomycin (all from Gibco, Grand Island, NY, USA). Then 4‐μg plasmid DNA in Opti‐MEM was mixed with lipofectamine LTX and PLUS transfection reagent (Invitrogen), and added to the cells. For cotransfection, 1‐μg FAM20C plasmid DNA and 4‐μg MEPE plasmid DNA were applied. After 48 hours, the supernatants were collected and centrifuged at 8,000*g* for 20 min.

His‐tagged recombinant MEPE was purified using a metal‐chelate affinity column (1 mL; QIAGEN, Valencia, CA, USA) charged with nickel ions. Bound protein was eluted with 250mM imidazole in PBS. The fractions containing MEPE were identified by Western blotting.

### Western blotting

Recombinant MEPEs were loaded onto NuPAGE 10% Bis‐Tris gels (Invitrogen), fractionated by SDS/PAGE, and electrophoretically transferred onto polyvinylidene difluoride membranes for immunodetection. The membranes were blocked in 2% Tween in Tris‐buffered saline before addition of a c‐*myc* monoclonal antibody (1 μg/mL) (9E10; Thermo Fisher Scientific, Waltham, MA, USA). MEPE was detected with alkaline phosphatase‐ (ALP‐) conjugated secondary immunoglobulins.

### Phospho‐imaging (pIMAGO) detection of protein phosphorylation

Protein phosphorylation was detected with the pIMAGO HRP Phosphoprotein Detection Kit (Tymora Analytical, West Lafayette, IN, USA). Proteins were separated by SDS/PAGE and electrophoretically transferred onto a polyvinylidene fluoride membrane. The membrane was blocked with 0.5% BSA and 0.1% polyamidoamine dendrimers. Next, the membrane was incubated with the pIMAGO Ti^2+^/biotin‐dendrimer reagent and washed with 50mM 2,5‐dihydrobenzoic acid in 0.1% trifluoroacetic acid. Avidin‐horseradish peroxidase was applied, and phosphoproteins were visualized with 3,3′‐diaminobenzidine. For dephosphorylation, 1‐μg MEPE was incubated with 30 mU of bovine ALP (Merck & Co., Kenilworth, NJ, USA) in 50mM ammonium bicarbonate overnight at 37°C.

### Purification of ASARM peptides

There were 20‐μg MEPE and MEPE‐(K526A/K532A) mutant expressed with and without FAM20C, which were digested with trypsin (Merck) using an enzyme‐to‐substrate ratio of 1:50 (w/w), in 50mM ammonium bicarbonate, at 37°C for 6 hours. The tryptic digests were separated by reverse‐phase high‐performance liquid chromatography (RP‐HPLC) on an Aeris C4 widepore column (Phenomenex, Torrance, CA, USA) connected to a Shimadzu HPLC System (Shimadzu, Kyoto, Japan). The peptides were separated at 40°C in 0.1% trifluoroacetic acid (TFA; buffer A) and eluted with a gradient of 60% acetonitrile in 0.1% TFA (buffer B). The gradient was developed over 59 min (0 to 5 min: 0% buffer B; 5 to 49 min: 0‐98% buffer B; 49 to 59 min: 98% buffer B) at a flow rate of 0.85 mL/min. The peptides were detected by measuring the absorbance at 226 nm and the resulting fractions were collected for analyses.

To identify fractions containing *myc*‐tagged peptides, 20 μL of each fraction were mixed with 80‐μL PBS and coated on 96‐well MaxiSorp immunoassay plates (Thermo Fisher Scientific) overnight at 4°C. The plate was extensively washed with PBS and blocked with 2% ovalbumin (Merck) in PBS for 1 hour at 37°C. All washes and dilutions after this were performed in PBS‐Tween [10mM disodium phosphate anhydrate (pH 7.2), 150mM NaCl, 0.05% (v/v) Tween 20]. Next, 0.25‐μg c‐*myc* monoclonal antibody (9E10; Thermo Fisher Scientific) were added to each well, followed by incubation with horseradish peroxidase‐conjugated polyclonal goat anti‐mouse IgG (DAKO, Carpentaria, CA, USA) (diluted 1:1000). Both antibodies were incubated for 1 hour at 37°C. Color development was obtained with TMB‐one substrate (Kem‐En‐Tec Diagnostics, Taastrup, Denmark), and the reaction was stopped by the addition of 0.2M H_2_SO_4_. Color intensity was measured at 450 nm using an ELISA reader (BioTek, Winooski, VT, USA). Fractions containing *myc*‐tagged peptides were further analyzed by MALDI‐MS. For dephosphorylation, 20 μL of HPLC fractions containing ASARM peptides were dried in a vacuum centrifuge and incubated with ALP as described above.

### Mass spectrometry

MALDI‐MS was performed using a Bruker Autoflex III instrument operated in linear mode and calibrated using Protein Calibration Standard I (Bruker Daltonics, Billerica, MA, USA). Samples for MS analyses were prepared by mixing the fraction to be analyzed with 2,5‐dihydroxybenzoic acid (10 mg/mL) in a 1:1 ratio directly on the MS‐target probe. The theoretical peptide masses were calculated using the GPMAW program (Lighthouse Data, Odense, Denmark).

LC–MS/MS analysis of trypsinized MEPE samples was performed on a QExactive+ mass spectrometer (Thermo Fisher Scientific) equipped with an EASY‐nLC 1000 (Thermo Fisher Scientific). All samples were micropurified using 3‐M Empore C18 discs (3M Co., Saint Paul, MN, USA) packed in pipette tips.^(^ ^26^ ^)^ The lyophilized samples were dissolved in 0.1% formic acid, injected on a trap column (2 cm × 100 μm inner diameter), and separated on an analytical column packed inhouse in a pulled emitter (15 cm × 75 μm inner diameter). All columns were packed with ReproSil‐Pur C18‐AQ 3‐μm resin (courtesy of Dr Marisch, Ammerbuch‐Entringen, Germany). Peptides were eluted directly into the mass spectrometer with a linear gradient from 5% to 35% solvent B (0.1% formic acid in acetonitrile) for 20 min with a flowrate of 250 nL/min, followed 10 min with 100% solvent B. The acquired raw data was converted to Mascot Generic Format using the Raw Converter (version 1.1.0.18). The generated peak lists were searched against the Swiss‐Prot database using an inhouse Mascot search engine (Matrix Science, London, UK). The following search parameters were used: *Homo sapiens*, trypsin, maximal two missed cleavages, carbamidomethyl as fixed modification, and oxidation and phosphorylation as variable modifications. Peptide tolerance was set to 10 ppm and MS/MS tolerance was set to 0.1 Da. MS/MS spectra of the targeted phosphorylated peptides were validated manually.

### Multiple sequence alignment

Sequence analyses were performed on MEPE from the following 14 mammalian species found in the UniProt database https://www.uniprot.org/ (UniProt release 2020_01): Human, *Homo sapiens* (Q9NQ76); chimpanzee, *Pan troglodytes* (A0A2I3ST89); Bornean orangutan, *Pongo pygmaeus* (D6C6N8); bovine, Bos tausrus (E1BG36); pig, *Sus scrofa* (I3LGA7); horse, *Equus caballus* (F6XRW9); sheep, *Ovis aries* (W5NXZ2); rabbit, *Oryctolagus cuniculus* (G1SG94); cat, *Felis catus* (A0A337S4X3); dog, *Canis lupus familiaris* (D6C6L9); guinea pig, *Cavia porcellus* (D6C6M4); mouse, *Mus musculus* (Q8K4L6); rat, *Rattus norvegicus* (Q9ES02); and dolphin, *Lipotes vexillifer* (A0A340WT16). Sequence alignment was performed with the UniProt website alignment tool.

## Results

**Fig 1:**
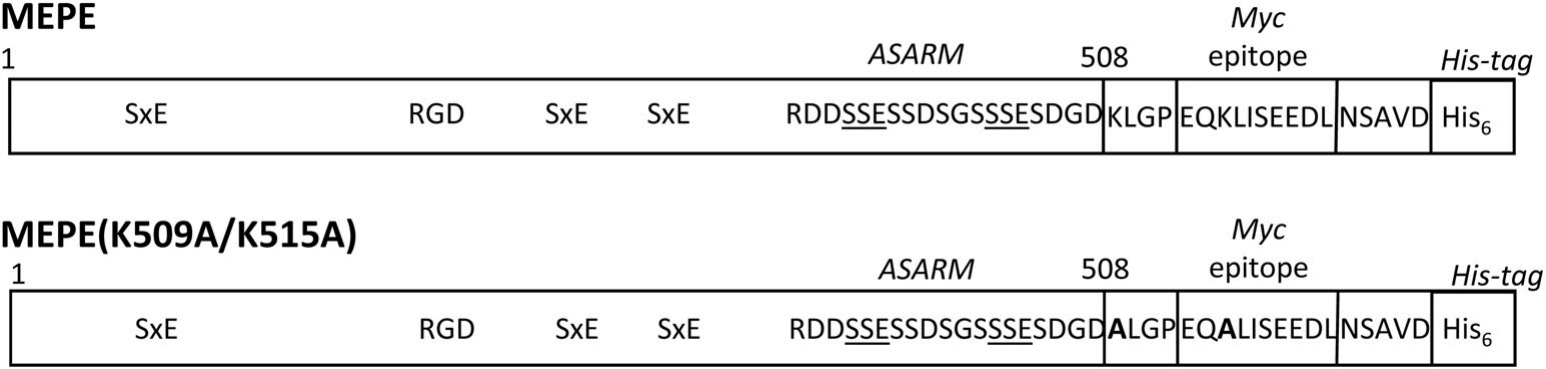
Schematic representation of the two matrix extracellular phosphoglycoprotein (MEPE) variants used in this study. The recombinant human MEPE proteins were expressed in HEK293T cells and contained a C‐terminal *myc*‐ and ‐His tag. The numbering of amino acids corresponds to the position in the MEPE sequence consisting of 508 amino acids. The integrin‐binding RGD sequence, serines located in the FAM20C recognition motif (Ser‐x‐Glu), and the acidic serine‐ and aspartate‐rich motif (ASARM) are indicated in the figure. The two lysine residues mutated to alanine in MEPE (K509A/K515A) are shown in bold.

**Fig 2:**
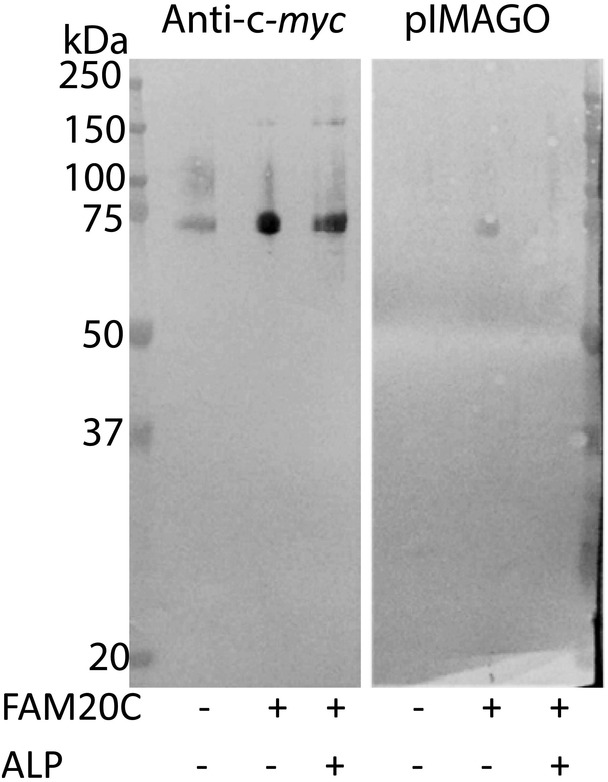
Western blot and phospho‐imaging (pIMAGO) staining of matrix extracellular phosphoglycoprotein (MEPE). MEPE expressed by HEK293T cells was analyzed by Western blotting with an anti‐c‐*myc* antibody or pIMAGO that stains phosphorylated proteins. Coexpression with FAM20C and dephosphorylation by alkaline phosphatase (ALP) are indicated.

To investigate the FAM20C‐mediated phosphorylation of MEPE, two recombinant forms of the MEPE protein (Fig. 1) were coexpressed with FAM20C in HEK293T cells. First, HEK293T cells were cotransfected with FAM20C and MEPE, and the resulting phosphorylation of MEPE was analyzed using pIMAGO, which specifically detects phosphorylated proteins. The pIMAGO reagent recognized MEPE coexpressed with FAM20C, but not MEPE expressed without the kinase (Fig. 2). Following treatment with ALP, the MEPE coexpressed with FAM20C was not detected in the pIMAGO assay, indicating FAM20C phosphorylation of MEPE. As control, an anti‐c‐*myc* antibody detected all MEPE forms (Fig. 2).

MEPE expressed alone, or coexpressed with FAM20C, in HEK293T cells was digested with trypsin, and the resulting peptides were characterized by LC–MS/MS. By this approach, 22 sites of phosphorylation (20 to 21 serine and 1 to 2 threonine residues) were identified in MEPE coexpressed with FAM20C (Table 1). Note, that this analysis did not include the ASARM sequence, as the C‐terminal part of MEPE was not detected in the LC–MS/MS analysis. MEPE expressed by HEK293T cells without coexpressed FAM20C only contained a single phosphorylated residue, Ser^43^ (data not shown). Spectral counts (listed in Table 1) depend on the ionization efficiency, as well as on the concentration of the peptides, and it can be used to estimate the abundance of a peptide and consequently the phosphorylation degree of a residue. Four phosphorylated residues, Ser^43^, Ser^222^, Ser^362^, and Ser^441^, had high spectral count numbers marking them as major FAM20C phosphorylation sites. The two peptides ^103^STGNKGFEDGDDAISK^118^ and ^366^RKEGSSDAAESTNYNEIPK^384^ were both found to contain a single phosphorylation, but the phosphorylated residue could not be unambiguously assigned to a specific residue between two potential phosphor accepting residues.

**Table 1:** LC–MS/MS Characterization of Phosphorylated Matrix Extracellular Phosphoglycoprotein (MEPE) Peptides

Peptide	P residue	ppm	Counts	Expect	Conservation (%)
^37^INQEL**S**SKENIVQER^51^	42	0.14	2	1,60E‐07	36
^36^RINQELS**S**KENIVQER^51^	**43**	0.9	39	1,10E‐08	21
^53^KDL**S**LSEASENK^64^	56	0.055	7	2,90E‐06	64
^52^KKDLSL**S**EASENK^64^	58	0.56	2	2,90E‐04	21
^54^DLSLSEA**S**ENKGSSK^68^	61	1	7	1,20E‐06	43
^54^DLSLSEASENKG**S**SK^68^	66	0.55	1	4,20E‐04	86
^103^ ***ST***GNKGFEDGDDAISK^118^	103 or 104	0.11	1	6,00E‐07	79,79
^158^NVLNIIPA**S**MNYAK^171^	166	2.74	3	4,30E‐05	36
^214^KIPSDFEG**S**GYTDLQER^230^	222	0.87	20	6,10E‐09	100
^231^GDNDI**S**PFSGDGQPFK^246^	236	0.06	1	5,60E‐05	86
^231^GDNDISPF**S**GDGQPFK^246^	239	0.36	6	4,90E‐08	100
^263^DIQTGFAGP**S**EAESTHLDTK^282^	272	0.92	1	5,40E‐07	79
^263^DIQTGFAGPSEAE**S**THLDTK^282^	276	1.02	7	7,00E‐12	71
^263^DIQTGFAGPSEAES**T**HLDTK^282^	277	2.75	3	1,50E‐06	50
^309^EADAVDV**S**LVEGSNDIMGSTNFK^331^	316	5.3	1	1,30E‐09	93
^337^EGNRVDAG**S**QNAHQGK^352^	345	0.69	1	2,70E‐06	100
^353^VEFHYPPAP**S**K^363^	**362**	0.67	30	4,30E‐05	93
^366^RKEG***SS***DAAESTNYNEIPK^384^	370 or 371	1.43	4	3,80E‐07	79,64
^414^GK**S**QGLPIPSR^424^	416	0.39	4	8,20E‐05	64
^432^NEMD**S**FNGPSHENIITHGR^450^	436	0.39	5	1,60E‐05	93
^432^NEMDSFNGP**S**HENIITHGR^450^	**441**	1.56	31	4,6E‐09	29
^432^NEMD**S**FNGP**S**HENIITHGR^450^	436,**441**	1.1	1	5,60E‐04	93,29
^467^GMPQGKG**S**WGR^477^	474	0.05	2	4,80E‐04	86

Note. The phosphorylated residues are underlined and shown in bold. If the phosphorylation had an equal possibility to be located on two different residues, the residue is shown in italics. The sequence position of phosphorylated residues located in the FAM20C recognition motif Ser‐x‐Glu is shown in bold. The ppm differences between the measured and theoretical calculated masses are listed. The spectral counts include all identified peptides containing the specific phosphorylated residue. The shown expect value is for the highest‐scoring peptide containing the residue. Conservation indicates how many of the analyzed mammalian MEPE sequences (Supplementary Fig. S1) contain the specific phosphorylated residue.

**Fig 3:**
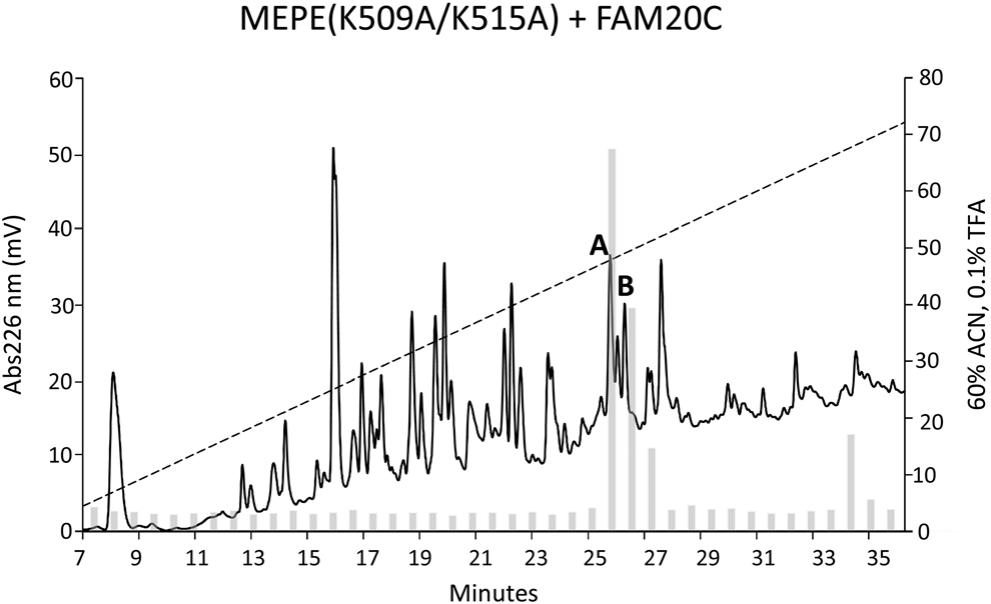
Reverse‐phase high‐performance liquid chromatography (RP‐HPLC) separation of trypsin‐digested MEPE(K509A/K525A) coexpressed with FAM20C. Peptides were separated by RP‐HPLC and eluted with a gradient of 60% acetonitrile in 0.1% trifluoroacetic acid (dashed line). The peptides were detected in the effluent by measuring the absorbance at 226 nm (solid line). Vertical bars show the relative amount of acidic serine‐ and aspartate‐rich motif (ASARM) and *myc*‐c containing peptides, as determined by an anti‐*myc* antibody as described in the Material and Methods section. Peaks A and B indicate the fractions with major anti‐*myc* antibody reactivity.

**Fig 4:**
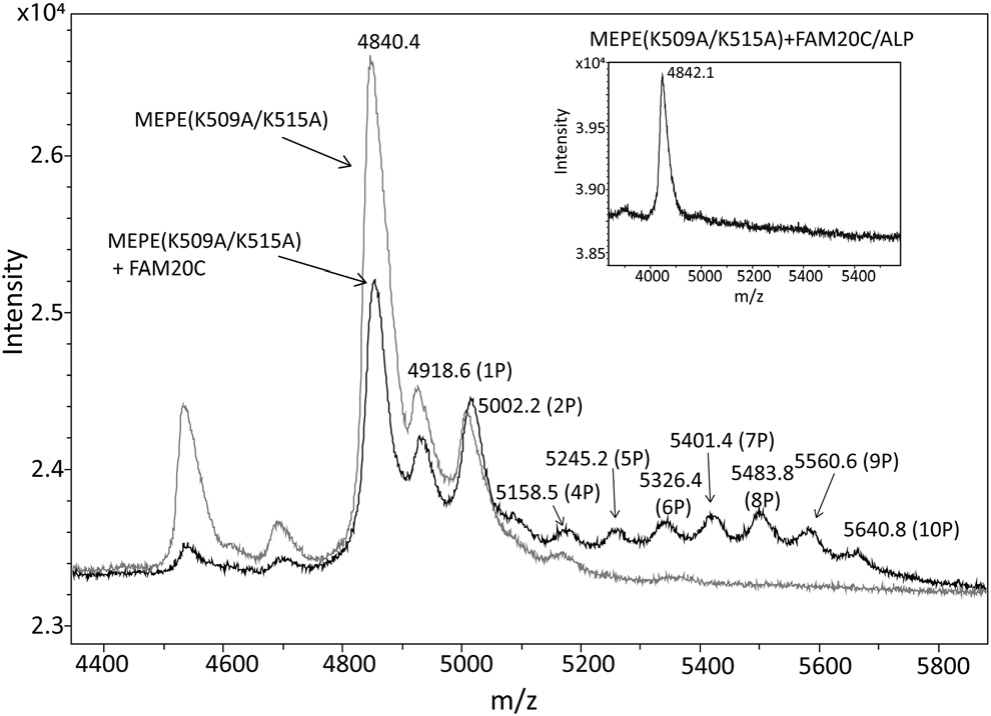
MALDI‐MS analysis of ASARM‐containing peptides. MEPE(K509A/K515A) coexpressed with FAM20C (black trace; peak A in Fig. 4) or without FAM20C (gray trace). The protonated average mass at *m/z* 4840.4 corresponds to the peptide ^489^RRDDSSESSDSGSSSESDGD^508^ALGPEQALISEEDLNSAVDHHHHHH containing the ASARM motif and the *myc*‐ and His‐tag. The other indicated masses correspond to different phosphorylated forms of the peptide denoted (P). The inset shows MALDI‐MS analysis of the ASARM‐containing peptide from MEPE(K509A/K515A) expressed with FAM20C after treatment with alkaline phosphatase (ALP). ASARM = acidic serine‐ and aspartate‐rich motif; MEPE = matrix extracellular phosphoglycoprotein.

**Fig 5:**
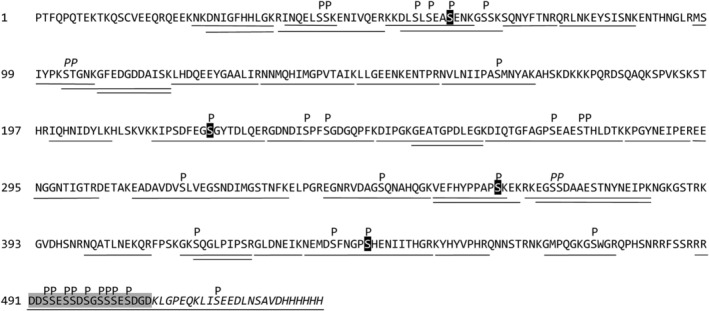
Localization of phosphorylated residues in human matrix extracellular phosphoglycoprotein (MEPE). Solid lines indicate all peptides characterized by LC–MS/MS or MALDI‐MS. P denotes identified phosphorylation and major FAM20C phosphorylation sites based on the intensity of the MS signal are highlighted in black. Phosphorylations with an equal possibility to be located at two different residues are shown in italics. The ASARM (acidic serine‐ and aspartate‐rich motif) is highlighted in gray and the *myc*‐ and His‐tag are shown in italics.

The LC–MS/MS analysis of the tryptic digest did not show any peptides containing the ASARM motif, probably attributable to poor ionization of this very acidic sequence. To overcome this, the residues Lys^509^ and Lys^515^ were mutated to alanine residues, which results in a larger C‐terminal tryptic peptide containing the ASARM motif as well as the *myc*‐epitope (see Fig. 1). The mutated MEPE(K509A/K515A) was coexpressed with FAM20C, digested with trypsin, and the resulting peptides were separated by RP‐HPLC. All fractions were subsequently incubated with an anti‐*myc*‐c antibody to identify fractions containing the *myc*‐epitope, and hence also the ASARM motif. Two major peaks (labeled *A* and *B* in Fig. 3) contained peptides reacting with the anti‐*myc‐c* antibody. In a parallel experiment, a *myc*‐c containing tryptic peptide from MEPE(K509A/K515A) expressed in HEK293T cells without FAM20C was purified (data not shown). The fractions were analyzed by MALDI‐MS to determine the phosphorylation degree of the ASARM motif (Fig. 4). Analysis of peak A showed an *m/z* value of 4840.4 Da corresponding to the mass of the peptide ^489^RRDDSSESSDSGSSSESDGD^508^‐ALGPEQALISEEDLNSAVDHHHHHH (MH^+^ 4838.8 Da). Several additional peaks with mass increments of approximately 80 Da were observed for MEPE(K509A/K515A) coexpressed with FAM20C. The highest *m/z* value at 5640.8 had a mass increment of approximately 800 Da corresponding to the mass of 10 phosphorylations (one phosphorylation was located in the *myc*‐tag, see Fig. 5). After treatment with ALP, peak A only showed one peptide mass at *m/z* 4842.1 corresponding to the unmodified peptide (inset in Fig. 4). Peak B in Fig. 3 also contained the ^489^Arg‐Asp^508^‐*myc*/His peptide, but with fewer phosphorylated residues (data not shown). MS analysis of the ^489^Arg‐Asp^508^‐*myc*/His ASARM peptide expressed without FAM20C also revealed some phosphorylations, but significantly less than when expressed together with FAM20C (Fig. 4). The total sequence coverage and resulting map of phosphorylations of MEPE are shown in Fig. 5.

**Fig 6:**
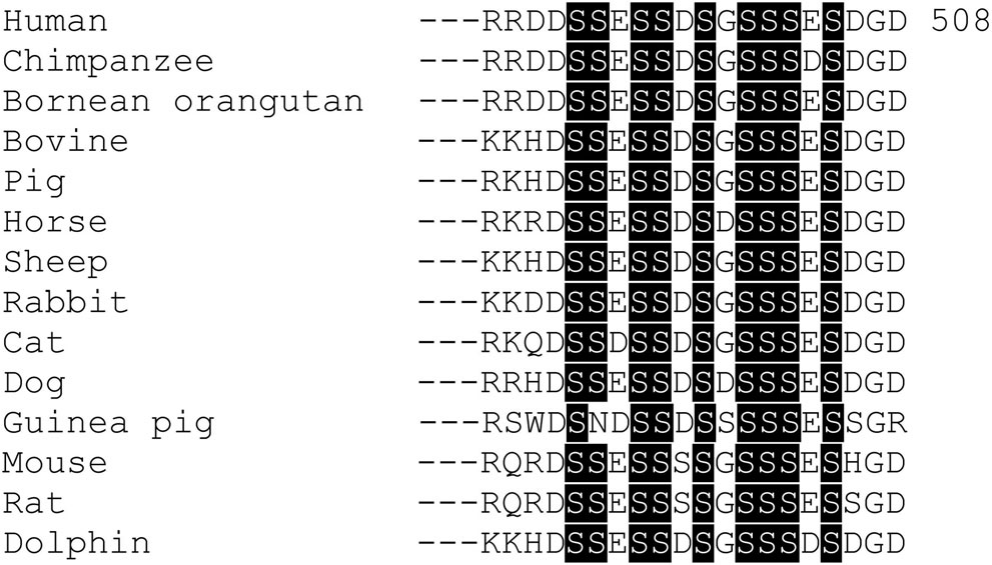
Sequence alignment of the acidic serine‐ and aspartate‐rich motif (ASARM) sequence in matrix extracellular phosphoglycoprotein (MEPE). Sequence alignment of 14 mammalian MEPE ASARM sequences from the UniProt database (UniProt release 2020_01). Phosphorylated residues identified in this study are highlighted in black.

Protein sequence alignment of mammalian MEPE sequences showed that Ser66, Ser236, Ser316, Ser362, Ser436, and Ser474 identified as phosphorylated in this study, are conserved in more than 80% of the aligned sequences, and that Ser222, Ser239, and Ser345 are conserved in all sequences (Table 1, Supplementary Fig. S1). Interestingly, all nine serines in the ASARM motif are fully conserved except for Ser494, which is only missing in the guinea pig sequence (Fig. 6, Supplementary Fig. S1).

## Discussion

The present study describes the phosphorylation of human MEPE coexpressed with FAM20C in kidney epithelial HEK293 cells, showing a remarkably high level of phosphorylation, similar to what we have shown previously for OPN.^(^ ^27^ ^)^ HEK293 cells have a very low endogenous expression of FAM20C,^(^ ^28^ ^)^ and the SIBLING proteins OPN, BSP, and MEPE expressed without FAM20C in HEK293 cells have previously been found not to be phosphorylated,^(^ ^23^ ^)^ thus demonstrating the specificity of this phosphorylation event for SIBLING proteins. Here, it was first shown that MEPE expressed in HEK293 cells without FAM20C was not recognized by the pIMAGO phosphorylation‐detecting reagent (Fig. 2), thus indicating that there was no (or very little) endogenous phosphorylation of MEPE by this cell line. After coexpression with FAM20C, 22 phosphorylation sites were identified in the MEPE sequence by LC–MS/MS analysis (not including the ASARM sequence). Spectral counts from the analyses indicated that the major FAM20C phosphorylation sites are Ser^43^, Ser^222^, Ser^362^, and Ser^441^. Ser^43^, Ser^362^, and Ser^441^ are located in Ser‐x‐Glu motifs, the canonical phosphorylation sequence of FAM20C: These three serine residues constitute all residues located in this motif outside of the ASARM sequence in MEPE. Ser^222^ is not located in a motif that is considered a FAM20C phosphorylation site. MEPE also contains four serine residues located in Ser‐x‐Asp motifs, sites that have been shown to be phosphorylated in another SIBLING protein, OPN.^(^ ^27^ ^)^ Of these sites, only Ser^239^ and potentially Ser^370^ were found to be phosphorylated by FAM20C, and the spectral counts indicate that they are minor sites of phosphorylation. Collectively, this emphasizes that FAM20C preferably phosphorylates serine residues in Ser‐x‐Glu motifs. Nineteen phosphorylated residues were located in motifs not complying with the canonical consensus sequence of FAM20C, Ser‐x‐Glu/pSer (Table 1). This finding is in line with other studies that have shown that FAM20C is capable of phosphorylating residues in a great variety of motifs.^(^ ^24^ ^)^ Of note, FAM20C can phosphorylate serine residues in an SxQxxDEE motif where the acidic residues in position *n + 5* to *n + 7* are critical determinants.^(^ ^29^, ^30^ ^)^ The two phosphorylated residues of MEPE, Ser^56^ and Ser^436^, are located in sequences with acidic residues in the *n + 5* to *n + 7* positions.

LC–MS/MS analysis showed that Ser^43^ was the only phosphorylated residue in MEPE without coexpressed FAM20C. Ser^43^ is located in a Ser‐x‐Glu motif, and the phosphorylation can most likely nevertheless be ascribed to the low endogenous expression of FAM20C by HEK293 cells.^(^ ^28^ ^)^ The phosphorylation was not detected by the pIMAGO reagent indicating that the level of phosphorylation is low (Fig. 2). In FAM20C KO mice, OPN has been shown to be still phosphorylated at FAM20C recognition motifs.^(^ ^31^ ^)^ This indicates that other kinases apart from FAM20C can phosphorylate the SIBLING proteins. In the present study, 22 phosphorylated residues were identified by LC–MS/MS in MEPE (not including the ASARM sequence) after coexpression with FAM20C, whereas only a single phosphoserine residue was identified when MEPE was expressed alone. The overexpression of FAM20C and MEPE in the HEK293 cells can potentially result in phosphorylation of residues that are not phosphorylated in vivo. However, the results strongly indicate that FAM20C is the primary kinase responsible for MEPE phosphorylation, as has been shown for other SIBLING proteins, particularly for OPN.^(^ ^22^, ^23^, ^24^ ^)^

The C‐terminal part of MEPE containing the ASARM peptide was not identified in the LC–MS/MS analysis most likely because of the acidic nature of the peptide. To identify and characterize peptides containing the ASARM motif, we expressed the MEPE(K509A/K515A) mutant with and without coexpression of FAM20C in HEK293 cells. The mutation of the two lysine residues made it possible to generate tryptic peptides of a size and charge that could be analyzed by MS. Moreover, the substitution of the lysine residues leads to tryptic peptides that contain the *myc*‐tag located just after the ASARM sequence (see Fig. 1). The ASARM‐containing peptide ^489^RRDDSSESSDSGSSSESDGD^508^ALGPEQALISEEDLNSAVDHHHHHH was efficiently phosphorylated by FAM20C, as MALDI‐MS analysis showed that it contained up to 10 phosphorylations after coexpression of MEPE with FAM20C (Fig. 4). The ^489^Arg‐Asp^508^‐*myc*/His peptide contains 11 serine residues, of which only four residues (one positioned in the *myc* epitope) conform to the FAM20C consensus sequence Ser‐x‐Glu/pSer. This indicates that the kinase can phosphorylate rows of consecutive serine residues in MEPE, even though they are not positioned in the consensus sequence. Likewise, FAM20C has been shown to phosphorylate polyserine stretches devoid of Ser‐x‐Glu/pSer motifs in phosvitins^(^ ^25^ ^)^ and serine residues in the ASARM of OPN not located in Ser‐x‐Glu motifs.^(^ ^23^ ^)^ The ^489^Arg‐Asp^508^‐*myc*/His peptide was also observed with two phosphorylations in MEPE expressed without FAM20C. This provides additional evidence for some endogenous FAM20C activity in HEK293 cells.

Ser^43^, Ser^222^, Ser^362^, and Ser^441^ were indicated to be major FAM20C phosphorylation based on spectral counts (Table 1). Sequence alignment showed that Ser^222^ and Ser^362^ were highly conserved (Table 1, Fig. S1), whereas Ser^43^ and Ser^441^ located in Ser‐x‐Glu motifs were only present in approximately one‐third of the aligned sequences. Other residues indicated as minor FAM20C target sites, Ser^66^, Ser^236^, Ser^239^, Ser^316^, Ser^345^, Ser^436^, and Ser^474^, are conserved in more than 80% of the aligned sequences. Only Ser^222^, Ser^239^, and Ser^345^ are conserved in all species. In comparison, other important motifs like N‐glycosylation sites and the integrin binding RGD‐sequence are conserved in approximately 40% to 60% and approximately 93% of the sequences, respectively (Supplementary Fig. S1). The conservation of these serine residues suggests that they may be important for MEPE function even if they are not located in the FAM20C recognition motif. In particular, the alignment highlights the importance of the ASARM, as all aligned sequences contained nine serine residues except guinea pig, where only Ser^494^ is missing (Fig. 6, Supplementary Fig. S1).

Phosphorylation of the MEPE‐ASARM peptide is a requirement for its ability to act as an inhibitor of mineralization,^11^, ^13^ ^)^ and for its involvement in phosphate homeostasis by binding and inhibiting the enzyme activity of PHEX.^(^ ^19^, ^21^ ^)^ In these previous studies, a synthetic phosphorylated ASARM peptide, RDDSSESSDSGpSSpSEpSDGD, was utilized. This synthetic phosphorylated ASARM peptide contains three phosphoserines, which we show are also phosphorylated by FAM20C, as all nine serine residues in the ASARM are. In our study, we have used a genetically altered construct to facilitate purification and MS identification of the tryptic ASARM peptide. Additional experiments are needed to confirm the phosphorylation pattern in vivo.

The conservation of all serine residues in the ASARM motif (Fig. 6) indicates that phosphorylation could influence the functionality of the potent MEPE‐ASARM peptide. For instance, the phosphorylation degree of the OPN‐ASARM peptide influences its activity, where it was shown that PHEX addition to bone cell cultures rescued inhibition of mineralization by the peptide when containing three phosphoserines, but not when containing five phosphoserines.^(^ ^17^ ^)^ Taken together, these findings imply that the extent of phosphorylation of SIBLING proteins is likely used as a fine‐tuning mechanism for a variety of functions in mineralized tissue biology. Paramount among these functions appears to be the strong influence of the phosphorylation status of SIBLING proteins such as MEPE and OPN in regulating extracellular matrix mineralization. Particularly noteworthy in these two cases of high phosphorylation for secreted proteins is that there is quite a spread (albeit with clusters) of phosphorylation sites along the full‐length of the proteins. This provides a means by which to distribute additional negative charge (beyond many Asp and Glu residues) maximally across proteins that are intrinsically disordered^(^ ^32^ ^)^ and seemingly designed by nature to be flexible and versatile polyelectrolytes,^(33^ ^)^ capable of binding to the many different mineral phases found in different types of organisms across many phyla in the animal kingdom. Phosphorylation of secreted proteins is a relatively new area of research as compared with our knowledge on intracellular kinase phosphorylating pathways, yet recent and forthcoming data align well with there being prominent roles for this posttranslational modification in the assembly and mineralization of extracellular matrices. Indeed, the importance of secreted‐protein phosphorylation in mineralized tissues can be recently exemplified by studies showing that mutation of even the single phosphorylation site (Ser^16^) in the major secreted enamel protein (amelogenin) results in faulty mineralization involving precursor‐phase transitions and disorganized and hypoplastic tooth enamel.^(^ ^34^ ^)^

In summary, our findings describe the first full characterization of MEPE phosphorylation. We show that coexpression in a eukaryotic cell line of the SIBLING protein MEPE and the kinase FAM20C results in up to 31 different residues in MEPE being phosphorylated, the major sites being Ser^43^, Ser^222^, Ser^362^, and Ser^441^. Moreover, the C‐terminal ASARM peptide of MEPE implicated in various mineralization functions contains nine serine residues that are maximally phosphorylated by FAM20C, further supporting the importance of this conserved, posttranslationally modified ASARM peptide in mineralized tissue biology.

## Disclosures

MDM currently has ongoing funding discussions with Ultragenyx regarding its research and clinical programs for the treatment of X‐linked hypophosphatemia. All other authors declare no conflicts of interest.

## Supporting information


**Fig. S1.** Sequence analyses were performed on MEPE from the following 14 mammalian species found in the UniProt database (UniProt release 2020_01). The phosphorylated residues identified in this study are highlighted in black and the integrin binding RGD sequence and sites for N‐glycosylation are highlighted in grey. Human, homo sapiens (Q9NQ76); chimpanzee, Pan troglodytes (A0A2I3ST89); Bornean orangutan, Pongo pygmaeus (D6C6N8); bovine, Bos tausrus (E1BG36); pig, Sus scrofa (I3LGA7); horse, Equus caballus (F6XRW9); sheep, Ovis aries (W5NXZ2); rabbit, Oryctolagus cuniculus (G1SG94); cat, Felis catus (A0A337S4X3); dog, Canis lupus familiaris (D6C6L9); guinea pig, Cavia porcellus (D6C6M4); mouse, Mus musculus (Q8K4L6); rat, Rattus norvegicus (Q9ES02); dolphin, Lipotes vexillifer (A0A340WT16).
