# Supplementary material for: FAM20C‐Mediated Phosphorylation of MEPE and Its Acidic Serine‐ and Aspartate‐Rich Motif
Source: JBMR Plus. 2020 Jun 26;4(8):e10378. doi: 10.1002/jbm4.10378 (PMC7422707; doi:10.1002/jbm4.10378)
Supplement: Supplementary file 1 — Fig. S1. Sequence analyses were performed on MEPE from the following 14 mammalian species found in the UniProt database (UniProt release 2020_01). The phosphorylated residues identified in this study are highlighted in black and the integrin binding RGD sequence and sites for N‐glycosylation are highlighted in grey. Human, homo sapiens (Q9NQ76); chimpanzee, Pan troglodytes (A0A2I3ST89); Bornean orangutan, Pongo pygmaeus (D6C6N8); bovine, Bos tausrus (E1BG36); pig, Sus scrofa (I3LGA7); horse, Equus caballus (F6XRW9); sheep, Ovis aries (W5NXZ2); rabbit, Oryctolagus cuniculus (G1SG94); cat, Felis catus (A0A337S4X3); dog, Canis lupus familiaris (D6C6L9); guinea pig, Cavia porcellus (D6C6M4); mouse, Mus musculus (Q8K4L6); rat, Rattus norvegicus (Q9ES02); dolphin, Lipotes vexillifer (A0A340WT16). [file JBM4-4-e10378-s001.pdf]

Figure S1

```
                                                  -17       1
Human               ------------------------MRVFCVGL-LLFSVTWAAP---TFQPQTEKTKQSCV  15
Chimpanzee          ------------------------MRVFCVGL-LLVSVTWAAP---TFQPQTEKTKQSCV
Bornean orangutan   ------------------------MRVFCVGL-LLFSVTWAAP---TFQPQTEKTKQSCV
Bovine              ------------------------MRIFCLEL-LLFSLTLAAP---TLQPQAEKKKQDYV
Pig                 ------------------------MRIVCLGL-FLFSLTWAAP---TFQPQAEKAKHDCV
Horse               MPETANPAEAARYSECKRPQRFSKMQIVCWGL-LLLSVTWAAP---ALQPQTEKTEQDCV
Sheep               ------------------------------------------------------------
Rabbit              ------------------------MPVIYIGL-LLLGVSWAAPVSMTFRPQTKETQRGCV
Cat                 ------------------------MQIVCLGL-LLFSVTWAAP---TFQPQTEKTKEDCV
Dog                 ------------------------MQTVCLGL-LLFSVTWAAP---TFQPQTGKTKDDCV
Guinea pig          ------------------------MQAVCVRLLLLFSVTWTAT-----------------
Mouse               ----------------MTPEGLMKMQAVSVGL-LLFSMTWAAPMP---------------
Rat                 ------------------------MQAVSVGL-FLFSMTWAAPKL---------------
Dolphin             ------------------------MRIISLGL-LLFSLTLAAP---TLQPQAEKMKQDTV

                                                                         42/43
Human               EEQR-------------------------------QEEK-NKDNIGFHHLGKRINQELSS  43
Chimpanzee          EEQR-------------------------------QEEK-NKDNIGFHHLGKRINQELSS
Bornean orangutan   EEQRITYKGHHEKHGHYVFKCVYMSPGKKNQTDVKQEEK-NKDNTGFHHLGKRINQELSP
Bovine              EEQRITYKGRHEKHGYYMFKYVSTSSGRKNQTDMK-EEK-NK-GIALPHSGKRSQ-EPAP
Pig                 EKQR------------------------------Q-EEK-NKGDIALPHSGKRE----AP
Horse               EEQRK-------------------------------EEK-NKDNIAVHVSGKGRNQEPAP
Sheep               -----------------MFKHVSTSSGRKNQTDVK-EEK-NK-GIALPHSGKRSQ-EPAP
Rabbit              EEQRMTYKGHHEKHGHYIFKCVYTLPGKKNQTNVKEEEKKDKENAALDHFDKRRNQQLSP
Cat                 EEQRQ-------------------------------EEK-KKDSIAHHNSGKRRNQEPAP
Dog                 EEQRITYKGHHEKHGYYIFKYVYTSSGRKNQTDIKQEEK-NKDNTALQNSGKRRNQEPAP
Guinea pig          ------------------------------------QEEKSEDDIAPAHFDKRRKQELSS
Mouse               ------------------------------------NED---------------------
Rat                 ------------------------------------NED---------------------
Dolphin             EEQRIMYKGHHAKHGYYVFKYVYTSSGRKNQTDAK-EEK-NKDNIALPHSGQRRN-QE--

                               56 58 61   66                                 103
Human               KENIVQERKKDLSLSEASENKGSSKSQNYFTNRQRLNKEYSISNKENTHNGLRMSIYPKS  103
Chimpanzee          KENIVQERKKDLSLSEASENKGSSKSQNYFTNRQGLNKEYSISNKENTHNGLRMSIYPKS
Bornean orangutan   KENIVQERKKDLSLSEASENKGSSKSQNYFTNRQRLNKEYSISNKENTHNGLRMSIYTKS
Bovine              KENIVLKKEEDLFLPRTNENNKSTKSQSLSGNKQSMNEDYIISSKENAHSDLKMSIYPES
Pig                 TENTGQEKGKNLSLPGNSENNKSTKAQNLIGNRKTMNEDQSIGNKENAQNDLKMAIYPKS
Horse               KENIAQEREKDLSLLGANENNQSSKSQTLFEDRQTVKEDYRISKKENARNDLKMSTYPES
Sheep               KENIVQKKEEDLFLPRTNENNKSTKSQNLSGNKQSMNEDYSISSKENAHSDLKMSIYPES
Rabbit              KDTA---QGRDLALLETIGKNRSVKSQTPSTNRQTLNEDLSVIT-TSVHNDREMSIYPEP
Cat                 KENIVQEREKSLSIVGANENNQSSKTQTPFENRQATNEDDTINNKENAHSDQKRYIYPES
Dog                 KENIAQEREKNLSILGASENKESSKTQTLFENIQTTNEVDSINNKENAHSDLKMSVYLES
Guinea pig          RENIVAEGEKMLSLFEASGNNRSSKSLHLSPDRQTLKEADRVIHKDQAGEHPMVPTYPES
Mouse               --------------------------------------RSSCGNQDSIHKDLAASVYPDP
Rat                 --------------------------------------GSSGGNQGNIHL---ASVKPEP
Dolphin             --PASQEKEKDVSLPGTNENNKSTKSQNLLENRQTMNKDYRISKKENAHNDLKTSIYPES

                   104
Human               TGNKGFEDGDDAISKLHDQEEYGAALIRNNMQHIMGPVTAIKLLGEENKENTPRNVLNII  163
Chimpanzee          TGNKGFEDGDDAISKLHDQEEYGAALIRNNMQHIMRPVTAIKLLGEENKENTPRNVLNII
Bornean orangutan   TGNKGFEDGDDAISKLHDQEEYGTALIRNNMQHIMGPVTAIKLLEEENKENTPRNVLNKI
Bovine              KGKHRAEDRDSAIRKLHSQEGYGAALIRNSMYHGMEPRTVTELLGGENKENKPRNVLGKI
Pig                 SENHKAEDRNSALSKLQDQEEYGAALIRNHMQHAMEPGAVIELLGEENKENKPRNVLSKI
Horse               TGNNGAKDGDDAVSKLHDQEEYSAHLLRKNMQHIMEPGTVVELLGEENKENKPRKVVSKI
Sheep               TGKHGAEDRDSAIRKLHNQEGYGAALIRNKMYHRMEPGTVIELLGKENKENKPRKVLGKL
Rabbit              TGDRGVEDGDDATGKFHDQEEYSTGLIGKNTQHTMGPMMVTGALGEENRKSKLRNVVRSI
Cat                 TGNNGVDDGDNAISKLRDQEEHGTALIRNNMQHIMEPGTVTELLAEENKENQHRNVLSKI
Dog                 PGNNGAEDKDNAINKSHDQEEYGTALIRNNMQHIMEPGTVTELLAEENKENKPRNILSKI
Guinea pig          TGPSGTKDGDSGMRSLHVQ-EYGTALLRNKAPAVTRPVTVSEWWEGQDSEKNPRNVPRNV
Mouse               TVDEGTEDGQGALLHPPGQDRYGAALLRNITQPVKSLVTGAELRREGNQEKRPQSVLSVI
Rat                 MVGKGTEGGRDAPLHLLDQNRQGATLLRNITQPVKSLVTGTEVQSDRNKEKKPQSVLSVI
Dolphin             TGNRGTEDGDNAVSKLHDQEEYGKALIRNNMHHIMEPGTVIELLREENKENKARNVLSKV

                      166
Human               PASMNYAKAHSKDKKKPQRDSQAQKSPVKSKST---HRIQHNIDYLKHLSKVKKIPSDFE  220
Chimpanzee          PASMNYAKAHSKDKKKPQRDSQAQKSPVKSKST---HRIQHNIDYLKHLSKVKKIPSDFE
Bornean orangutan   PASMNYAKAHSKDKKKPQRDSQAQKSPVKSKST---HRIQHNIDYLKHLSKVKKIPSDFE
Bovine              PADANYAKAPSQL-KNHQRDSQAQNIPVKSKST---HLTQHNMDYLKQLPKVKKIPSDFE
Pig                 PAAANYAKAPSNIKKNHPRGSQAQNIPVKSRSP---HRLQHNTDYLKQLPKVKKVPSDFE
Horse               PAGASYAKGPSKDKKSHQKDPQAQNIPVKSKST---YRIQHNTDYLKQLPKVKKIPSDVE
```

```
Sheep                PADANHAKAPSRL-KNHQRDSQAQNIPVKSKTT---HLIQHNMKRLKQLPKVKKIPSDFE
Rabbit               PEGIHYAKIHSKEKRNHQRDSQVQSSPIKSKSI---RHIQHNADYLKQLPKVKKVSRDFE
Cat                  PASVNYVKVPSKDRKNYQRDPQAQNIPVKSKST---RLTQYNTDYSKQLPKLKKIPSDFE
Dog                  PASVNYVKVPSKNRKNYQRDPQAQNIPVKSKST---HHTQHNIDYPKQLQKVKKIPSDFE
Guinea pig           PADVNYAKARSQGKKRHQPGAGGRSNPGRSTGA---GHTQCSAHCQTQLSNAPRIPRDFE
Mouse                PADVNDAKVSLKDIKNQESYLLTQSSPVKSKHTKHTRQTRRSTHYLTHLPQIKKTPSDLE
Rat                  PTDVHNTNDYSEDTENQQRDLLLQNSPGQSKH---TPRARRSTHYLTHLPQIRKILSDFE
Dolphin              PADANYAKASSKIKKNHQRDSQAQNIPVKSKST---HRIQHNMDYLKQLPKAKKVSSDFE

                      222          236 239                              272 276/277
Human                GSGYTDLQERGDNDISPFSGDGQPFKDIPGKGEATGPDLEGKDIQTGFAGPSEAESTHLD 280
Chimpanzee           GSGYTDLQERGDNDISPFSGDGQPFKDIPGKGEATGPDLEGKDIQTGFAGPSEAESTHLD
Bornean orangutan    GSGYTDLQERGDNDISPFSGDGQPFKDIPGKGEATGPDLEGKDIQTGFAGPSEAESTHLD
Bovine               GSGYPDLQGRGDNDLSPFSGDGPPFKDISGKGDAIDPDRKGTDIQTELSIPSETETTSPD
Pig                  GSGYPDLQGRGDNDISPFSGDGPPFKDLSGKGEATGPDREGTDIQTEFSSPSEAETIHPD
Horse                GSGYPDLQERGDNDISPFSGDGQPFKDISGKGEAIGPDPEGADVQTEFSGPSEAETSHPD
Sheep                GSGDPDLQGRGDNDLSPFSGDGPPFKDISGKGDAIDPDREGTDIQTELPSPSETETTSPD
Rabbit               GSGYT---GRGDNDITPFSGDGQPSKDIPRKGGVIGPDPGGIDVQTGFSGPRKAEAIDPD
Cat                  GSGYPDLQGRGDNDISPFSGDGQPFKDISGKEEPIGPDLKGTDIQTEFSGPSEA-----D
Dog                  GSGYPDLQERGDNDISPFSGDGQPFKDISGKGEPIGPDLKGTDIQTESSGPREADTISPD
Guinea pig           GSGYTGAQGRGDNDVSPFSGDGQPFQDIPDKG------------QTGLLVPGEAETSDTD
Mouse                GSGSPDLLVRGDNDVPPFSGDGQHFMHIPGKGGA-GSGPESST-SRPLSGSSKAEVIDPH
Rat                  DSASPDLLVRGDNDVPPFSGDGQHFMHTPDRGGAVGSDPESSA-GHPVSGSSNVEIVDPH
Dolphin              GSGYPDLQGQED-NISPFSGDGPPFKDISGKGDAIRPDREGTDIQTEFSSPSEAETINPD

                                                    316
Human                TKKPGYNEIPEREENGGNTIGTRDETAKEADAVD-VSLVEGSNDIMGSTNFKELPGREGN 339
Chimpanzee           TKKPGYNEIPEREENGGNTIGTRDETAKEADAVD-VSLVEGSNDIMGSTNFKELPGREGN
Bornean orangutan    TKKPGYNEIRKREENGGNTIGTRDETVKEADAVD-VSLVEGSNNIMGSTNFKELPGREGN
Bovine               AKGPGYNEIPEKERNGRKTTGTRGKTAQEAN-TD-VSLVEGSNDIIGGTDFKKLPGQEGN
Pig                  ARGPGSNEIPEKEGNGRDTIGTRGKTAKEANGAD-VSLVEGGNGITGSTDFKKLPGKEGN
Horse                ARGPGYNEIPEEEENGGNAIGTRDGTAKEANAAG-VSLVEGSNDIIGSTDFKKLPGKEGN
Sheep                AKGPGYNEIPETERNGRKTTGTRGKTAQEANTAD-VSLVEGTNDIIGGTDFKKLPGQEGN
Rabbit               TKGPGYNEIPEREENGGDAFGTRDQTAKEATD---VSLAKGSNDIIGSTNFKKLPGKEGN
Cat                  ARGPGYNEIPEKEENGRNTIGTRDETRKEANTADVSLVVEGSNDIIGSTNFKELPGKEGN
Dog                  ARRPGYNEIPEREESGGSTTGTRDETRKEASTD--VSLVEGSNDIIGSTNFKELPGKEGN
Guinea pig           RRGLGPNETPETDGGRDNVLVARVGTEKEAGAA-VPSLGEGSSDLMGSTNFRDLPGTQGN
Mouse                MSGLGSNEIPGREGHGGSAYATRDKAAQGAGSAG-GSLVGGSNEITGSTNFRELPGKEGN
Rat                  TNGLGSNEIPGREGHIGGAYATRGKTAQGAGSAD-VSLVEGSNEITGSTKFRELPGKEGN
Dolphin              ARGPGYNEIPEKEGNGGNTIGTRDETAQKANAAD-VSLVEGNNNIIGSTNFKKLSGKEGN

                          345              362     370/371
Human                RVDAGSQNAHQGKVEFHYPPAPSKEKRKEGSSDAAESTNYNEIPKNGKGSTRKGVDHSNR 399
Chimpanzee           RVDAGSQNAHQGKVEFHYPPAPSKEKRKEGSSDATESTNYNEIPKNGKGSTRKGVDHSNR
Bornean orangutan    RVDAGSQNAHQGKVEFHYPPAPSKEKRKEGSSDATESTNYNEIPRNGKGSTRKVVDHSNR
Bovine               RVHGGSQDAHQGEIEFHYPHVPSKDQRKDGSRDGAEGTNYNEIPPNGKGSSRKGTQHSTR
Pig                  RVDGSSQNAHQGKIEFHYPHAPSKDKRKGGSSDGAESTNYNEIPKYGKGSSRKGPGPSDR
Horse                RVDAGSQNAHQGQVEFHYPDPPSKEKRKEGTSDVTESTNYNEVPKNGKGSGRKSPEHSNR
Sheep                RVDGGSQDAHQGEIEFHYPHVPSKDKRKHGSSDGAESTKYNEIPKNGKGSSRKSTEHSAR
Rabbit               RVDADSQNAHQGKVEFHFPQAPSQGKRKEGSRDATPSTNYNEIPKNGKGSSRKGTGHSDR
Cat                  RVDASSQNAHQGKVEFHYPHPPSKEKKKEGSSDIAESTDYNEIPKNGKGTSRKGTEHSNR
Dog                  RVGANSQNAHQGKVEFHYPFPPSKEKKAEGSSDVAESTNYNEIPKNGKGSSRKDTEHSNR
Guinea pig           RVDMGSQNAHRGKVELHYPSVPFQGQTKGGGGVASKSVAHNEIPKHGKGSARKATEPSGR
Mouse                RINAGSQNAHQGKVEFHYPQVASREKVKGGV-EHAGRAGYNEIPKSSKGSSSKDAEESKG
Rat                  RVDASSQNAHQGKVEFHYPQAPSKEKVKGGSREHTGKAGYNEIPKSSKGGASKDAEESKG
Dolphin              RVDGGSENAHQGEIEFHYPHAPTEDKRKDGGSDGAEGT--NEIPKNGKGSSRKDTEHSKR

                                416                 436  441
Human                NQATLNEKQRFPSKGKSQGLPIPSRGLDNEIKNEMDSFNGPSHE-NIITHGRKYHYVPHR 458
Chimpanzee           HQATLNEKQRFPSKGKSQGLPIPSRGLDNEIKTEMDSFNGPSHE-NIITHGRKYHYVPHR
Bornean orangutan    NQATLNEKQRFPSKGKSQGLPIPSRGLDNEIKTEMDSFNGPSHE-NIITHSRKYHYVPHR
Bovine               YQEPSKEKQRFPSKSEGQGQLIPPRGLDNEIKHEIGSHNGPNNEGTITTHSRKNHYVPHR
Pig                  SQVTSSEKQSFPSKGKGQGQLTPSRGLDNNIKNEMGSHSDPNNEGTIITHGRKNHDVHYR
Horse                NQVTSNEKQRFPGKSKSQGRFTPSRGLDHEIKNEIGSHNVPNNEGT-ITHSRKYHYVPHG
Sheep                NQEPSKEKQRFPSKSKGQGQLIPSHGLDNEIKNEISSHNGPNNEGTIITHSRKNHYVPHR
Rabbit               NQVNVHENQRFSAKGKSQDLLSPSRGLDNEMKNEIGSHGGPNNRGNTITHSRKNHYVPHP
Cat                  NQAISNEKQRFPSKGKSQGRLVPSRDLDNEVKNEIGSHNGLNNEGTIITHNRRNHYVPHR
Dog                  NKVISTGKQRFPTKGKSQSQLVPSHDLDNEIKNEIGSHNGLNNEETIITQSRKNHYVPHR
Guinea pig           NQAALSEKHQFFSRGESQRGIIPPRGLDNEIKNEISSHHGPHSEGNGVTHSRRNLQASRG
Mouse                NQLTLTASQRFPGKGKSQGPALPSHSLSNEVKSEENHY----------VF--------HG
Rat                  NQVTLTESQRFPGKGKGQS----SHSLGNEVKSEEDSSNSLSREGIAIAH--------RR
Dolphin              NQVTSNEKQRFPSKGKGQGQFIPSRGLDNEIKNEIGSHNGPNNEGTIITHSRKNYSVPHR
```

```
                                               474                       493-505
Human                          QNNSTRNKGMPQGKGSWGR-QPHSNRRFSSRRRDDSSESSDSGSSSESDGD 508
Chimpanzee                     QNSSTWNKGMPHGKGSWGR-QPHSNRRFSSRRRDDSSESSDSGSSSDSDGD
Bornean orangutan              QNNSTRNKGMPHGKGSWGR-QSHSNRRFSSRRRDDSSESSDSGSSSESDGD
Bovine                         QNNYAWNKGGSQSKGTWGYRNSHSNRRFRPPKKHDSSESSDSGSSSESDGD
Pig                            QNNYTRSKGMSQRKGSWGYRKPPSNRRFHPPRKHDSSESSDSGSSSESDGD
Horse                          QNNSSGNKGMAQRKGPWDYRKPHPNRSFSHPRKRDSSESSDSDSSSESDGD
Sheep                          QNNYTRNKGGSQSKGTWGYRSSHSNRRFRPPKKHDSSESSDSGSSSESDGD
Rabbit                         QDNEAWNSGVPQRKGLWGYRRSHSNRSFRLRKKDDSSESSDSGSSSESDGD
Cat                            Q-NSTRNKGVPQRKGSWGYRKPLSSRRFRPPRKQDSSDSSDSGSSSESDGD
Dog                            Q-NSTWNKGVPKRKGSWGYRKPHSRRRVSPPRRHDSSESSDSDSSSESDGD
Guinea pig                     QNDTVGGKAGAQRRGSWGHRRPHSHRGFSSPRSWDSNDSSDSSSSSESSGR
Mouse                          QNNLTPNKGMSQRRGSWPSRRPNSHRRAST-RQRDSSESSSSGSSSESHGD
Rat                            TSHPTRNRGMSQRRGSWASRRPHPHRRVST-RQRDSSESSSSGSSSESSGD
Dolphin                        QNNSMQNKGVSQSKGSWGYRKPHSNRRFHPPKKHDSSESSDSGSSSDSDGD
```

Sequence analyses were performed on MEPE from the following 14 mammalian species found in the UniProt database (UniProt release 2020_01). The phosphorylated residues identified in this study are highlighted in black and the integrin binding RGD sequence and sites for N-glycosylation are highlighted in grey.

Human, homo sapiens (Q9NQ76); chimpanzee, Pan troglodytes (A0A2I3ST89); Bornean orangutan, Pongo pygmaeus (D6C6N8); bovine, Bos tausrus (E1BG36); pig, Sus scrofa (I3LGA7); horse, Equus caballus (F6XRW9); sheep, Ovis aries (W5NXZ2); rabbit, Oryctolagus cuniculus (G1SG94); cat, Felis catus (A0A337S4X3); dog, Canis lupus familiaris (D6C6L9); guinea pig, Cavia porcellus (D6C6M4); mouse, Mus musculus (Q8K4L6); rat, Rattus norvegicus (Q9ES02); dolphin, Lipotes vexillifer (A0A340WT16).
